# Reflections of Two Parallel Pathways between the Hippocampus and Neocortex in Transient Global Amnesia: A Cross-Sectional Study Using DWI and SPECT

**DOI:** 10.1371/journal.pone.0067447

**Published:** 2013-07-05

**Authors:** Young Ho Park, Jae-Won Jang, Youngsoon Yang, Jung Eun Kim, SangYun Kim

**Affiliations:** 1 Department of Neurology, Seoul National University College of Medicine, Seoul, Korea; 2 Clinical Neuroscience Center, Seoul National University Bundang Hospital, Seongnam, Korea; 3 Department of Neurology, Seoul Veterans Hospital, Seoul, Korea; Centre Hospitalier Universitaire Vaudois Lausanne - CHUV, UNIL, Switzerland

## Abstract

**Objectives:**

Two parallel pathways have been proposed between the hippocampus and neocortex. Recently, the anterior and posterior hippocampus showed distinct connectivity with different cortical areas in an fMRI study. We investigated whether the two parallel pathways could be confirmed in patients with transient global amnesia (TGA) which is a natural lesion model of a perturbation of the hippocampus. In addition, we evaluated the relationship between the location of the hippocampal lesion and various clinical variables.

**Methods:**

A consecutive series of 37 patients were identified from the TGA registry database of Seoul National University Bundang Hospital. Based on the location of the diffusion-weighted imaging (DWI) lesion along the anterior-posterior axis of the hippocampus, they were divided into the following three groups: head (n = 15), body (n = 15) or tail (n = 7). To evaluate which cortical regions showed hypoperfusion according to the location of the DWI lesion, their SPECT images were compared between two groups using statistical parametric mapping. We performed hierarchical cluster analysis to group demographic and clinical variables, including the location of the DWI lesion, into clusters.

**Results:**

Statistical parametric mapping analyses revealed that more anterior DWI lesions were associated with hypoperfusion of the anterior temporal and frontal areas, whereas more posterior lesions were associated with hypoperfusion of the posterior temporal, parietal, occipital and cerebellar areas. The difference was most prominent between the group of hippocampal lesions on the head and tail. Hierarchical cluster analysis demonstrated that vomiting was related to female gender and hippocampal head lesions, whereas vascular risk factors were related to male gender and hippocampal body lesions.

**Conclusions:**

We confirmed the parallel pathways between the hippocampus and neocortex with DWI and SPECT images of patients with TGA. Patients with hippocampal head lesions and body lesions were clustered within different groups of clinical variables.

## Introduction

Transient global amnesia (TGA) is a syndrome of sudden-onset anterograde and retrograde amnesia which is not associated with other neurological deficits and disappears in less than 24 hours [1]. Although its exact pathogenesis is still not completely understood, hippocampal CA1 neurons are thought to be mainly involved in the pathophysiology of TGA [1]. In many patients with TGA, focal hyperintense diffusion-weighted imaging (DWI) lesions have been detected in the CA1 field of the hippocampal formation [2].

Since the hippocampal formation is connected with cortical areas [3], cortical neuronal activity might be influenced by hippocampal DWI lesions [4]. Most studies investigating regional cerebral blood flow in TGA via single photon emission computed tomography (SPECT) have noted mesiotemporal hypoperfusion with or without the concomitant involvement of various cortical, subcortical and cerebellar structures [4]–[14].

With respect to the connection between the hippocampus and neocortex, two parallel pathways have been proposed [3], [15], [16]. Recently the anterior and posterior hippocampus showed distinct connections with different cortical areas in an fMRI study [16]. The location of hypoperfusion in TGA might be potentially determined by the site of the DWI lesions along the anterior-posterior axis of the hippocampus.

In this study, we evaluated whether the two parallel pathways between the hippocampus and neocortex could be confirmed in TGA which is a natural lesion model of a perturbation of the hippocampus. Patients with TGA within 7 days from onset were grouped based on whether the DWI lesion was located at the head, body or tail of the hippocampus [17]. To investigate which cortical regions showed hypoperfusion according to the location of the DWI lesion, their SPECT images were compared between groups using statistical parametric mapping (SPM). In addition, we performed cluster analysis to find the relationship between the location of the DWI lesion and various clinical variables.

## Methods

This was a cross-sectional study of patients with TGA based upon a TGA registry database. A consecutive series of patients who visited Seoul National University Bundang Hospital within 7 days of symptom onset and fulfilled criteria for TGA between January 2008 and June 2011 were identified from the registry database. The diagnostic criteria for TGA were based on patient history and physical examination and included a bedside mental status examination. The criteria were: (i) presence of anterograde amnesia (e.g., asking repetitive questions or exhibiting temporal disorientation) that was witnessed by an observer, (ii) no clouding of consciousness or loss of personal identity, (iii) cognitive impairments limited to amnesia (e.g., lack of symptoms such as inability to recognize faces or common objects, difficulty thinking of common words while speaking or uncharacteristic mood change), (iv) no focal neurologic signs or epileptic features, (v) no recent history of head trauma or seizures and (vi) resolution of symptoms within 24 hours [18]. Patients who also had brain SPECT within 7 days of onset were included. We then selected patients who showed 1- to 5-mm punctate hyperintense lesions in the lateral portion of the hippocampus, probably around the CA1 field, on DWI [19]. We excluded patients who had extra-hippocampal structural lesions that caused perfusion abnormalities. Patients’ demographics, clinical profiles and imaging data were obtained directly from the registry database or through medical record review. MRI and SPECT images were read by board-certified neuroradiologists and nuclear medicine physicians, respectively. A paired t-test was used to evaluate whether scanning timing in respect to the chronology of each individual attack was different between DWI and SPECT. We compared patients who were included and excluded with respect to demographics and clinical characteristics using Student’s *t*-test for continuous variables. We used either Pearson’s chi-square test or Fisher’s exact test, as appropriate, for the categorical variables.

We divided the study population into the following three groups based on the site of the DWI lesion along the anterior-posterior axis: (i) hippocampal head lesion (lesions confined to the hippocampal head or concomitantly in both the hippocampal head and body), (ii) hippocampal body lesion (lesions confined to the hippocampal body) and (iii) hippocampal tail lesion (lesions confined to the hippocampal tail or concomitantly in both the hippocampal tail and body) [17]. Patients with simultaneous lesions of the hippocampal head and tail were excluded. Demographics and clinical characteristics among the groups were compared using the Fisher's exact test for categorical variables. Demographic variables were also compared using the Mann-Whitney U test for two groups (head and tail, head and body, body and tail) or the Kruskall-Wallis test for three groups (head, body and tail) for continuous variables.

### Imaging Parameters

MRI was usually performed within a few hours after arriving at the hospital on either a 1.5-Tesla unit (Intera; Philips Medical Systems, Best, the Netherlands) or a 3-Tesla unit (Intera Achieva; Philips, Best, the Netherlands) with a sensitivity encoding (SENSE) head coil. The MRI protocol included DWI, T1- and T2-weighted imaging; fluid-attenuated inversion recovery imaging; conventional gradient-echo imaging in the transverse plane; T1-weighted imaging in the sagittal plane; 3D time-of-flight angiography of the intracranial region; and contrast enhanced angiography of the neck region [19]. DWI was performed again at day 3 post-onset with the same imaging parameters. Single-shot spin-echo echo-planar imaging was used for DWI using the following parameters: matrix, 128×128 interpolated to 256×256; field of view, 220 mm; repetition time, 9,400 ms for 1.5 Tesla and 5,000 ms for 3 Tesla; echo time, 66 ms for 1.5 Tesla and 59 ms for 3 Tesla; SENSE factor, 2; number of acquisitions, 4; b value, 2,000 s/mm^2^; and section thickness, 3 mm.

SPECT images were obtained using a triple-head gamma camera (Trionix Triad; Trionix Research Laboratory, Inc., Twinsburg, OH) equipped with a low-energy, fan-beam collimator. Patients were instructed to close their eyes in a dimly lit room with minimal background noise. Scanning was initiated 10 minutes after an intravenous injection of 15 mCi of Tc-99m-ethylcysteine dimer. Data were acquired in a 128×128 matrix with a voxel size of 1.78×1.78×1.78 mm and then reconstructed by filtered back-projection using a Butterworth filter (cutoff frequency 0.6 cycle per cm, order 8) to reduce statistical noise. We also performed correction for tissue attenuation.

### SPM Analysis of Regional Perfusion

SPECT images were analyzed using SPM5 (Institute of Neurology, University College London, London, UK) [20], which was implemented using Matlab 7.8 (The MathWorks Inc., Natick, MA). The mean voxel intensity across all slices of the imaging volume was calculated. Each voxel was then thresholded at 80% of the mean intensity to eliminate background noise and partial volume effects at the edge of the brain. Each SPECT scan was then spatially normalized to the SPECT template provided by SPM5 using an affine geometric transformation. These images were smoothed using an isotropic Gaussian kernel of 16 mm full-width at half maximum. Then, a proportional scaling was applied to remove the effect of differences in global activity. After normalization and smoothing, statistical comparisons between two groups based on the site of the DWI lesion (head vs. tail, tail vs. head, head vs. body, body vs. head, body vs. tail, tail vs. body, left vs. right, right vs. left) were performed on a voxel-by-voxel basis using *t* statistics to generate SPM (*t*) maps. We investigated hypoperfusion areas at a threshold of *P*<0.005 (uncorrected) and an extent threshold of 100 voxels. For purposes of visualization, the significant voxels were projected onto the ch2better template included with MRIcron (http://www.mccauslandcenter.sc.edu/mricro/mricron/).

### Classification of Patients with TGA using Multivariate Analysis

Patients with TGA can be classified into groups based upon heterogeneities in pathophysiology [21]. To cluster demographic and clinical variables, including the location of the DWI lesion, we carried out a two-stage analysis procedure on the data by means of multiple factorial analysis (MFA) and hierarchical cluster analysis (HCA) [22], [23]. Because HCA is an exploratory method, the selection of the variables for the clustering classification is a critical problem [21]. We tried to resolve this problem by preceding HCA with MFA to select only pertinent variables for inclusion in HCA.

MFA distinguishes variables that explain the main part of the total variance [23]. It aims to reduce dimensionality with the least possible loss of information. We used non-linear optimal scaling transformation for our MFA [24]. We then performed HCA on the resulting variables. HCA aims to sort a set of objects into groups such that the objects in the same group are more similar to each other than to the objects in other groups [22]. We used Yule’s Q, a summary measure of pairwise association, based on the conditional odds ratio [25]. For the measure of distance between two groups, we used the unweighted pair group method with arithmetic mean [26]. One patient who had only imaging data without a detailed medical record available for review was excluded from MFA and HCA.

All statistical analyses were performed using the PASW statistical software version 19.0 (IBM Corp., Somers, NY), except for the SPM analysis. Because all data were analyzed anonymously, a waiver of informed consent was obtained from the Seoul National University Bundang Hospital Institutional Review Board, which approved this study.

## Results

We identified 88 TGA patients who arrived at the hospital within 7 days of symptom onset. Fifty-seven patients also had brain SPECT within 7 days of onset. Among these 57 patients, 41 patients had relevant hippocampal lesions on DWI during the study period. Four patients were excluded for the following reasons: old infarction in the temporal lobe with stenosis of the posterior cerebral artery (n = 1) and concomitant lesions of the hippocampal head and tail (n = 3). The study population consisted of the remaining 37 patients (Table S1). There was no hyperperfusion on visual rating of SPECT images. Time of onset to SPECT was significantly longer than time of onset to DWI with a mean difference of 13.36 hours (*P* = 0.031). The included patients had a longer duration of TGA and a higher incidence of dizziness compared with the excluded patients (Table S2).

**Figure 1:**
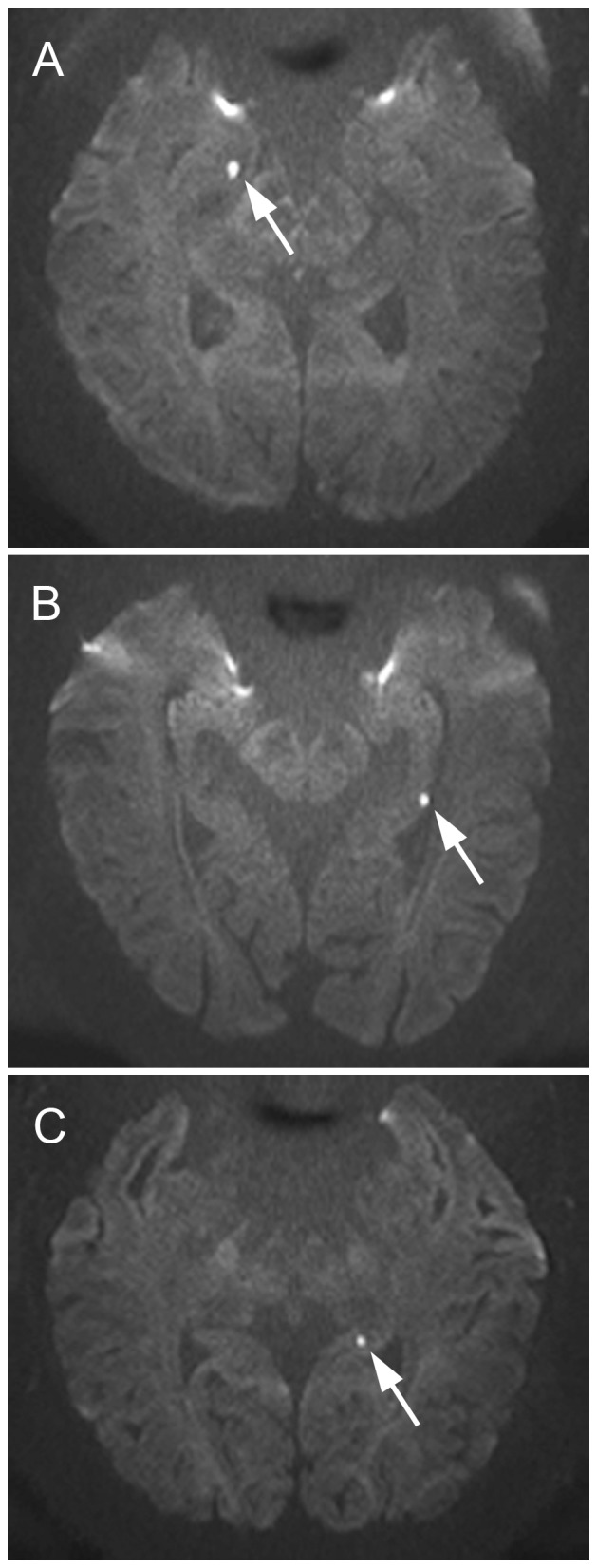
Examples of hippocampal head, body and tail lesion on diffusion-weighted imaging. The punctuate hyperintense lesions in the hippocampal head (A), body (B) and tail (C) were indicated with white arrows on axial diffusion-weighted imaging.

The baseline characteristics of the study population were summarized according to the site of the DWI lesion along the anterior-posterior axis of the hippocampus (Table 1). Respective examples of hippocampal head, body and tail lesion on DWI were demonstrated (Figure 1). In paired-group comparisons, the following variables were significantly different at *P*<0.05: the frequency of vomiting as a precipitating factor and nausea as an associated symptom in patients with hippocampal head lesions was higher than in patients with body lesions; the global amnesia lasted longer in patients with tail lesions than in patients with head lesions.

**Table 1:** Comparison of the baseline characteristics based on the site of DWI lesion.

	Hippocampal headlesion (n = 15)	Hippocampal bodylesion^a^ (n = 15)	Hippocampaltail lesion (n = 7)	P value^b^
**Age in years, median (IQR)**	59 (52–66)	62 (56–66)	60 (57–63)	0.654
**Men**	3 (20.0%)	6 (40.0%)	2 (28.6%)	0.649
**Precipitating factor**				
**Physical stress**	6 (40.0%)	7 (50.0%)	4 (57.1%)	0.753
**Emotional stress**	6 (40.0%)	8 (57.1%)	3 (42.9%)	0.683
**Vomiting**	5 (33.3%)	0	0	0.016
**Duration of TGA in hours, median (IQR)** c	5 (3–7)	6.5 (5.75–10.5)	7 (6.5–13)	0.079
**Associated symptoms**				
**Headache**	5 (33.3%)	3 (21.4%)	4 (57.1%)	0.277
**Dizziness**	3 (20.0%)	1 (7.1%)	0	0.501
**Nausea**	5 (33.3%)	0	0	0.016
**None**	7 (46.7%)	10 (71.4%)	3 (42.9%)	0.375
**Hypertension**	4 (26.7%)	6 (42.9%)	1 (14.3%)	0.411
**Diabetes**	1 (6.7%)	2 (14.3%)	0	0.588
**Hyperlipidemia**	3 (20%)	6 (42.9%)	4 (57.1%)	0.186
**Migraine**	0	3 (21.4%)	0	0.141
**Laterality of DWI lesion**				0.159
**Left**	4 (26.7%)	6 (40.0%)	1 (14.3%)	
**Right**	6 (40.0%)	8 (53.3%)	2 (28.6%)	
**Bilateral**	5 (33.3%)	1 (6.7%)	4 (57.1%)	
**Time of onset to DWI in hours, median (IQR)**	49 (7–72)	50 (10–72)	71 (9.5–72)	0.937
**Time of onset to SPECT in hours, median (IQR)**	48 (29–96)	45 (28–68)	49 (40–70)	0.902

Values are number (%) unless indicated.

Abbreviations: DWI = diffusion-weighted imaging; IQR = interquartile range; TGA = transient global amnesia.

a For one patient from group with hippocampal body lesions, only data concerning age, gender, laterality of DWI lesion, time of onset to DWI and time of onset to SPECT were available.

b P values were obtained using the Kruskall-Wallis test or the Fisher's exact test as appropriate.

c When patients were able to explain the reasons for their hospitalizations and to form new memories, we considered their episode of TGA to be over.

**Figure 2:**
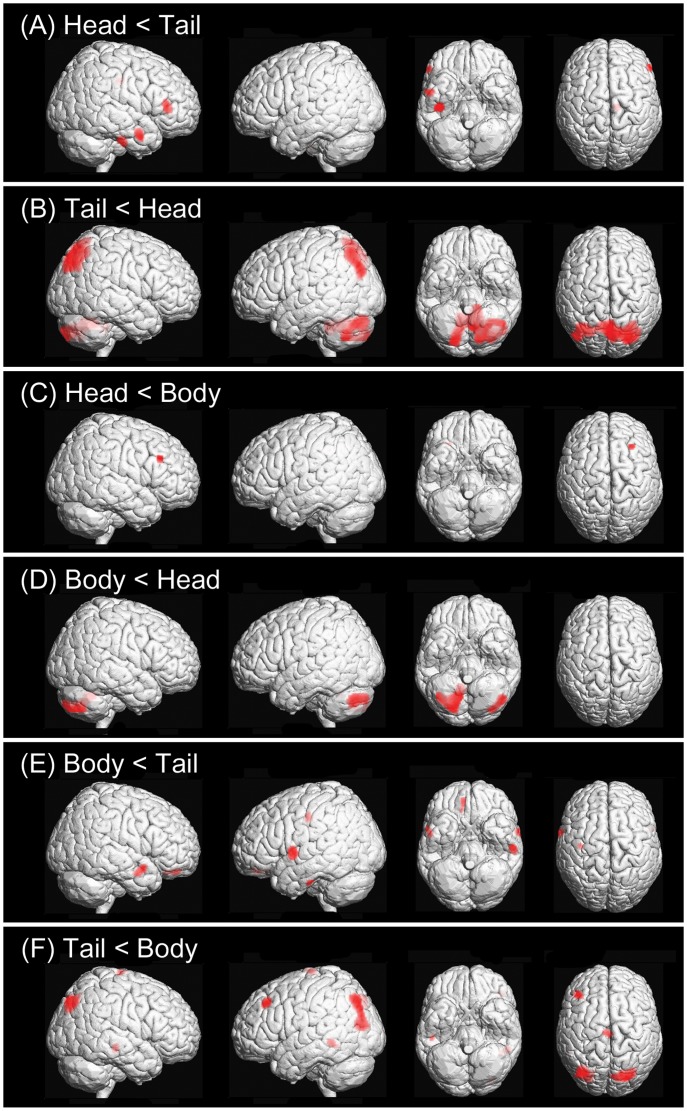
Areas of hypoperfusion in relation to the longitudinal location of the hippocampal lesion. Perfusion deficits in patients with hippocampal lesions on head vs. tail (A), tail vs. head (B), head vs. body (C), body vs. head (D), body vs. tail (E), and tail vs. body (F) were shown. The hypoperfusion areas (red color) were displayed on rendering images at a threshold of *P*<0.005 uncorrected, *k* = 100.

Perfusion was decreased in the inferior frontal and anterior temporal areas and the cingulate gyrus of patients with hippocampal head lesions compared with patients with tail lesions (Figure 2A, Table 2). Compared with patients with head lesions, patients with tail lesions showed perfusion deficits in the parieto-occipital and cerebellar areas (Figure 2B, Table 2). Blood flow in patients with head lesions was reduced in the middle frontal areas relative to patients with body lesions (Figure 2C, Table 2). Patients with body lesions had hypoperfusion in the cerebellar area compared with patients with head lesions (Figure 2D, Table 2). Perfusion was decreased in the frontal and anterior temporal areas and the postcentral gyrus in patients with body lesions compared with patients with tail lesions (Figure 2E, Table 2). Compared with patients with body lesions, patients with tail lesions showed hypoperfusion in the parieto-occipital and posterior temporal areas and in the middle frontal gyrus (Figure 2F, Table 2).

**Table 2:** Regions of perfusion deficit based on the site of hippocampal lesion along the anterior-posterior axis (*P*<0.005, uncorrected, *k* = 100).

Variables	Regions	BA	Stereotaxic coordinates			T value	Cluster size
			x	y	z		
Head<Tail	Rt. fusiform gyrus	20	−42	−18	−30	4.52	435
	Rt. middle temporal gyrus	21	−54	2	−24	3.26	320
	Rt. inferior frontal gyrus	45	−58	34	14	3.20	188
	Rt. middle cingulate gyrus	23	−12	−20	40	3.05	144
Tail<Head	Rt. Precuneus	7	−4	−66	62	5.61	5,089
	Rt. superior occipital gyrus	7	−28	−76	44	4.75	
	Lt. superior parietal gyrus	7	32	−64	52	4.50	
	Lt. cerebellar uvula	n/a	6	−54	−32	4.97	4,169
	Lt. cerebellar uvula	n/a	12	−44	−36	4.87	
	Cerebellar vermis	n/a	−2	−54	−30	4.47	
Head<Body	Rt. middle frontal gyrus	46	−30	28	32	3.33	105
Body<Head	Lt. crus cerebelli	n/a	46	−68	−38	3.76	318
	Lt. crus cerebelli	n/a	35	−80	−34	3.31	
	Lt. crus cerebelli	n/a	25	−82	−42	2.80	
	Rt. cerebellar uvula	n/a	−10	−54	−32	3.74	816
	Rt. cerebellar pyramis	n/a	−15	−58	−48	3.54	
	Rt. cerebellar pyramis	n/a	−40	−55	−50	3.24	
Body<Tail	Lt. Rolandic operculum	48	68	4	2	4.34	186
	Lt. postcentral gyrus	6	38	−14	42	3.86	202
	Lt. inferior temporal gyrus	58	58	−18	−36	3.76	104
	Rt. superior frontal gyrus	20	−12	48	−20	3.74	409
	Rt. rectus gyrus	11	−14	32	−20	2.98	
	Rt. middle temporal gyrus	21	−54	0	−22	3.30	144
	Rt. temporal pole	38	−50	10	−14	3.12	
Tail<Body	Lt. angular gyrus	7	36	−70	44	4.78	1,386
	Lt. middle occipital gyrus	19	28	−75	15	4.59	
	Lt. middle occipital gyrus	19	38	−75	30	3.21	
	Rt. superior occipital gyrus	19	−22	−78	40	4.55	829
	Lt. middle frontal gyrus	46	46	36	40	3.89	177
	Rt. middle temporal gyrus	20	−52	−26	−10	3.53	117
	Lt. middle temporal gyrus	21	52	−42	−4	3.32	202
	Lt. paracentral lobule	6	4	−16	80	3.22	147

Abbreviations: DWI = diffusion-weighted imaging; BA = Brodmann area; n/a = not available.

Patients with left hippocampal lesions showed perfusion deficit in the bilateral parietal areas relative to patients with right hippocampal lesions (Figure S1). Perfusion was decreased in the bilateral frontotemporal areas of patients with right hippocampal lesions compared with patients with left hippocampal lesions (Figure S1). Cerebral hypoperfusion was not lateralized according to the side of the hippocampal lesions.

**Figure 3:**
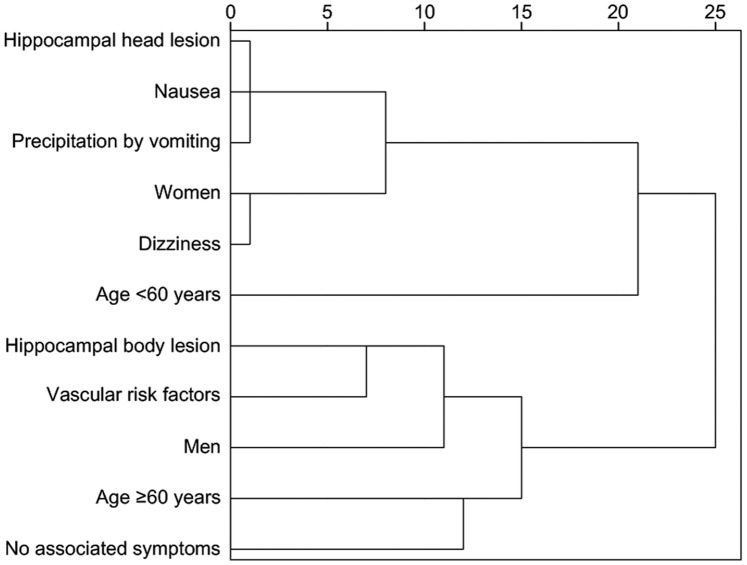
Dendrogram based on the hierarchical cluster analysis. Four principal clusters were observed: (i) women with symptoms of nausea and dizziness, patients with hippocampal head lesions, and patients who were induced by vomiting; (ii) patients aged <60 years; (iii) men with vascular risk factors and patients with hippocampal body lesions; and (iv) patients aged ≥60 years and patients without associated symptoms.

To classify the clinical features associated with the site of the DWI lesion, the following variables were collected initially: location of the DWI lesion (head, body, tail), gender, age (<60, ≥60 years), precipitating events (physical stress, emotional stress, vomiting), associated symptoms (headache, dizziness, nausea and none) and risk factors (hypertension, diabetes, hyperlipidemia, migraine and vascular risk factors). Vascular risk factors were defined to include hypertension, diabetes, hyperlipidemia, heart disease and smoking. Two dimensions explained 34.05% of the total variance in MFA. The fit of the model was evaluated with Cronbach’s alpha coefficient (0.729) and was acceptable [27]. The variables with the highest coefficient loadings (higher than 0.2) in at least one of the two dimensions were the location of the DWI lesion (head and body), gender, age, precipitation by vomiting, associated symptoms (dizziness, nausea and none) and vascular risk factors (Table S3). These variables were then included in HCA. The dendrogram showed four principal clusters (Figure 3). Patients with the hippocampal head lesions were grouped together with women, symptoms of nausea and dizziness and precipitation by vomiting. Patients with the hippocampal body lesions were grouped together with men and vascular risk factors.

## Discussion

Tract-tracing studies in rodents and monkeys have revealed the two parallel pathways through which unimodal and multimodal inputs reach the hippocampal formation [3]. Information from the anterior association regions, such as the medial and orbital frontal cortices, is segregated into the perirhinal cortex (PRC) and then into the lateral entorhinal area (LEA), whereas information from the posterior associational regions, such as the retrosplenial cortex, is segregated into the parahippocampal cortex (PHC) and then into the medial entorhinal area (MEA) [3], [28], [29]. The LEA is interconnected with the ventral (analogous to anterior hippocampal formation in primates) CA1 field and the subiculum, and the MEA is interconnected with the dorsal (analogous to posterior hippocampal formation in primates) CA1 field and the subiculum in rats [16], [30].

In humans, an fMRI study has shown that the PRC is preferentially connected with the anterior hippocampus, whereas the PHC is preferentially connected with the posterior hippocampus [16]. This anterior-posterior connectivity gradient is more robust in the CA1 field, where the DWI lesions have been detected in TGA, and in the subiculum than in the CA2/CA3/dentate gyrus region. The PRC shows preferential connectivity with an anterior temporal and frontal cortical network, and the PHC shows preferential connectivity with a posterior medial temporal, parietal, and occipital network [16].

In the current study, we compared the locations of perfusion deficits based on the site of the hippocampal lesion along the anterior-posterior axis in patients with TGA. An SPM analysis revealed that more anterior DWI lesions were associated with hypoperfusion of the frontal and anterior temporal areas on SPECT, whereas more posterior lesions were associated with hypoperfusion of the parietal, posterior temporal, occipital and cerebellar areas. These differences were most prominent between the two groups of patients with lesion on the head and tail of the hippocampus. Our results mirrored the previously reported parallel pathways between the human hippocampal formation and the neocortex [16].

In our patients, both of more left DWI lesions and more right DWI lesions were not associated with hypoperfusion of the lateralized region, but associated with hypoperfusion of the bilateral cortical areas. A previous fMRI study also showed that each hemispheric seed of the PRC and PHC was functionally correlated not only with the ipsilateral cortical areas, but also with the contralateral cortical areas [16].

Evidence for a functional differentiation between the anterior and posterior hippocampus has been suggested, but the topic is still controversial [31], [32]. It has been shown that the anterior hippocampus was related to episodic memory encoding, whereas the posterior hippocampus was associated with episodic memory retrieval [31]. However, consistent differences in mnemonic processes have not been reported [32].

Our HCA results revealed prototypical groups, reflecting the possibility of different pathophysiological mechanisms. Precipitation by vomiting was related to female gender and hippocampal head lesion, whereas vascular risk factors were related to male gender and hippocampal body lesions. It was previously suggested that the etiology of TGA could be an ischemic event due to arterial thromboembolism in patients with vascular risk factors or due to venous ischemia in patients with Valsalva-like activities before symptom onset [33]. The argument for the existence of two different mechanisms was supported by the finding that TGA patients with jugular vein valve incompetence showed a higher frequency of Valsalva-like maneuvers as precipitating factors and a lower frequency of vascular comorbidities than those in TGA patients without jugular vein valve incompetence [34].

These findings may be explained by structural characteristics of the hippocampal vasculature. First, the middle and the posterior hippocampal arteries, which supply the hippocampal body and tail, occasionally form a continuous arterial arcade [17]. The arcade and its branches arising at right angles may be related to the particular vulnerability of hippocampal tissue to hypoperfusion due to a sudden fall in blood pressure [17], [35]. Hence, the body and tail of the hippocampus may be more prone to ischemic injury in conditions with vascular risk factors. Second, venous flow in the hippocampal head runs only into the inferior ventricular vein, whereas the flow in the hippocampal body and tail runs into both the inferior ventricular vein and the medial atrial vein via the longitudinal superficial venous arches [17]. Therefore, the head of the hippocampus may be more susceptible to venous congestion due to the limited outflow tracts available.

Although most patients with TGA showed regional hypoperfusion [4]–[14], in a few patients in whom SPECT was performed during or immediately after the episode of TGA, there was transient hyperperfusion of the medial temporal or occipito-cerebellar areas [10], [36], [37]. This discrepancy between the results of previous SPECT studies may be due to different timings of the SPECT scans with respect to the chronology of each individual attack [37]. In our study, all patients were scanned after their recovery from amnesia and the median duration of time of onset to SPECT was 48 hours. Transient hyperperfusion, on the other hand, indicates that migraine-related mechanisms or epileptic phenomena might be involved in the pathogenesis of TGA in some patients [37].

Several limitations should be noted regarding this research. First, detailed neuropsychological assessments were unavailable and, therefore, were not included in the analysis. The pathophysiologic mechanisms responsible for the phenomenon of TGA could be elucidated more clearly by evaluating whether neuropsychological profiles are correlated with the location of regional hypoperfusion. Second, we could not perform SPM comparisons between TGA patients and normal controls because we did not have normal SPECT data available for use. The actual extent of hypoperfusion caused by TGA could be better evaluated by comparisons with normal controls rather than by comparisons between groups with hippocampal lesions in different sites. Third, there was a time gap between MRI and SPECT scanning in our retrospective study. Hippocampal DWI lesions were already detectable in the acute phase of the TGA, were most prominent 3 days after onset and completely diminished 10–14 days after onset [2], [19]. Regional hypoperfusion on SPECT in the acute phase of the TGA was gradually resolved during the following days and was normalized 15 days after onset [7], [9], [12]. In our study, a few days after TGA attack the DWI lesions still reflect the initial phase of TGA. In contrast, hypoperfusion on SPECT might be slightly diminished compared to the amount of hypoperfusion during the attack. Fourth, the significance levels were not corrected for multiple comparisons in SPM due to the small sample size. To reduce the false positive identification rate, we combined a two-sample *t*-test with a cluster filter of 100 voxels [38]. Fifth, there is the possibility of selection bias because the included patients had a longer duration of TGA and a higher rate of dizziness. Finally, this is a single-center study, which limits the generalizability of the study results.

In this study, we confirmed differential connectivity of the hippocampus with the neocortex along the anterior-posterior axis with DWI and SPECT images of patients with TGA. Understanding of this connectivity dissociation may help us grasp the pathophysiology of other neurological disorders that affect both the hippocampus and the neocortex, such as Alzheimer’s disease.

## Supporting Information

Figure S1 Areas of hypoperfusion in relation to the laterality of the hippocampal lesion.
(PDF)

Table S1 Clinical characteristics of the patients.
(DOC)

Table S2 Comparison of baseline characteristics between the included and excluded patients.
(DOC)

Table S3 Coefficient loadings of variables for each dimension in MFA (multiple factorial analysis).
(DOC)
