# Supplementary material for: Reflections of Two Parallel Pathways between the Hippocampus and Neocortex in Transient Global Amnesia: A Cross-Sectional Study Using DWI and SPECT
Source: PLoS One. 2013 Jul 5;8(7):e67447. doi: 10.1371/journal.pone.0067447 (PMC3702497; doi:10.1371/journal.pone.0067447)

Figure S1. Areas of hypoperfusion in relation to the laterality of the hippocampal lesion.

Perfusion deficits in patients with hippocampal lesions on left vs. right (A) and right vs. left (B) were shown. The hypoperfusion areas (red color) were displayed on rendering images at a threshold of $P < 0.005$ uncorrected, $k = 100$.

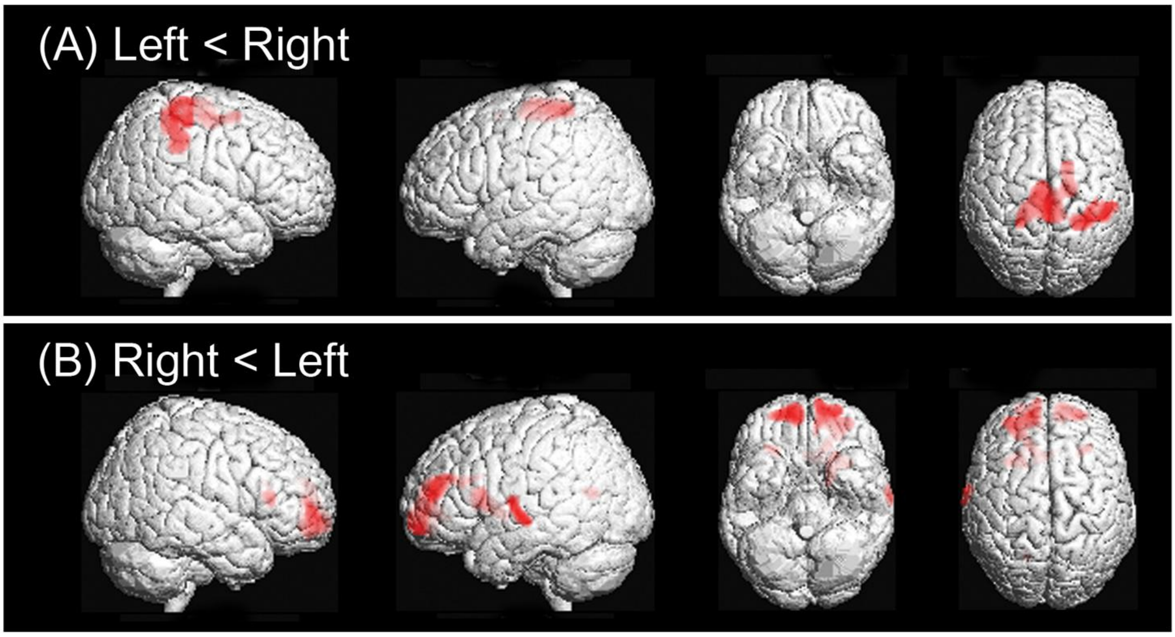

Supplement: Figure S1 — Areas of hypoperfusion in relation to the laterality of the hippocampal lesion. (PDF) [file pone.0067447.s001.pdf]
