# Supplementary material for: Reflections of Two Parallel Pathways between the Hippocampus and Neocortex in Transient Global Amnesia: A Cross-Sectional Study Using DWI and SPECT
Source: PLoS One. 2013 Jul 5;8(7):e67447. doi: 10.1371/journal.pone.0067447 (PMC3702497; doi:10.1371/journal.pone.0067447)
Supplement: Table S1 — Clinical characteristics of the patients. (DOC) [file pone.0067447.s002.doc]

**Table S1. Clinical characteristics of the patients.**

| No. | Age  (years) | Sex | Duration of TGA^a^ (hours) | Duration of retrograde amnesia | Circumstances of onset | Associated symptoms | Past medical history | Lesion of hippocampus on DWI | Time of onset to DWI (hours) | Visual rating of SPECT | Time of onset to SPECT (hours) |
| --- | --- | --- | --- | --- | --- | --- | --- | --- | --- | --- | --- |
| 1 | 59 | F | 6 | Days | Housework | None | HT | Left head, left body | 6 | Left temporal hypoperfusion | 26 |
| 2 | 52 | M | 16 | Days | After getting up in the morning | None | None | Right head | 6 | Normal | 34 |
| 3 | 51 | F | 11 | Months | After swimming | Headache | None | Left head | 7 | Normal | 29 |
| 4 | 67 | F | 6 | Days | Vomiting | Headache, nausea | HT, HL | Right head | 88 | Left frontoparietal hypoperfusion | 110 |
| 5 | 59 | F | 12 | Hours | Farm work | Headache, dizziness | None | Left head | 72 | Left temporal hypoperfusion | 73 |
| 6 | 71 | F | 3 | Hours | Cooking | None | HT, DM, HL | Right head | 72 | Normal | 71 |
| 7 | 51 | M | 4 | Hours | After showering | None | None | Right head, left body | 9 | Right temporal hypoperfusion | 5 |
| 8 | 66 | M | 6 | Hours | Vomiting | Nausea | None | Left head, right body | 49 | Right temporal hypoperfusion | 96 |
| 9 | 61 | F | 3 | Hours | Conversation | Headache | None | Bilateral head, right body | 31 | Right temporal hypoperfusion | 50 |
| 10 | 42 | F | 4 | Hours | Vomiting after undergoing endoscopy | None | None | Right head, left body | 68 | Bilateral temporal hypoperfusion | 48 |
| 11 | 66 | F | 3 | Days | Vomiting | Nausea, dizziness | HT, HL | Left head | 5 | Left temporal hypoperfusion | 11 |
| 12 | 57 | F | 7 | Years | After showering | None | None | Bilateral head | 71 | Bilateral temporal hypoperfusion | 138 |
| 13 | 59 | F | 5 | Days | Washing dishes | None | None | Right head | 12 | Right temporal hypoperfusion | 30 |
| 14 | 61 | F | 0.5 | Months | Vomiting | Nausea, dizziness | None | Right head | 89 | Left temporal, right frontotemporal hypoperfusion | 115 |
| 15 | 55 | F | 5 | Years | Bed rest due to headache | Headache, nausea, paresthesia, | None | Right head | 73 | Right temporal hypoperfusion | 45 |
| 16 | 59 | F | 16 | Days | Sporting | Headache | HT, HL | Right tail | 71 | Left temporal hypoperfusion | 67 |
| 17 | 60 | F | 6.5 | Days | Conversation | Headache | HL | Bilateral tail | 72 | Normal | 49 |
| 18 | 63 | F | 4 | Days | Clearing away the snow | None | None | Left tail | 72 | Right temporal hypoperfusion | 43 |
| 19 | 39 | M | 13 | Days | Skiing | None | None | Right tail | 10 | Right temporal hypoperfusion | 70 |
| 20 | 60 | F | 7 | Days | After crying | None | None | Left tail, right body | 5 | Normal | 40 |
| 21 | 68 | M | 12 | Days | After napping | Headache | HL | Right tail, left body | 9.5 | Right temporal hypoperfusion | 24 |
| 22 | 57 | F | 6.5 | Months | After getting up in the morning | Headache | HL | Left tail, right body | 74 | Bilateral temporal hypoperfusion | 74 |
| 23 | 50 | F | 5 | Hours | Swimming | dizziness | HL | Right body | 72 | Right temporal hypoperfusion | 10 |
| 24 | 54 | F | 21 | Hours | After dinner | Headache | HT, migraine | Left body | 70 | Right parietal hypoperfusion | 94 |
| 25 | 72 | F | 6 | Months | Conversation | Headache | HL | Right body | 6 | Normal | 45 |
| 26 | 63 | F | 5 | Days | Housework | Chilling sense | None | Bilateral body | 3 | Bilateral temporal hypoperfusion | 68 |
| 27 | 67 | M | 12 | Hours | After crying | None | HT, DM | Right body | 7 | Right frontotemporal hypoperfusion | 21 |
| 28 | 50 | M | 6 | Days | Conversation | Headache | DM, HL | Right body | 67 | Right temporal hypoperfusion | 66 |
| 29 | 62 | F | 8 | Days | After getting up in the morning | None | migraine | Left body | 72 | Left temporal hypoperfusion | 28 |
| 30 | 66 | F | 1 | Days | After showering | None | HT | Right body | 24 | Right temporal hypoperfusion | 45 |
| 31 | 56 | M | 10 | Hours | Climbing the mountain | None | HT | Left body | 10 | Bilateral temporal hypoperfusion | 28 |
| 32 | 67 | M | 7 | Years | Post-coital | None | HL | Left body | 72 | Left temporal hypoperfusion | 105 |
| 33 | 58 | M | 7.5 | Years | Sporting | Headache | HL | Left body | 50 | Normal | 24 |
| 34 | 63 | F | 6 | Hours | Conversation | None | None | Right body | 72 | Normal | 67 |
| 35 | 64 | M | 6 | Hours | Operating heavy vehicles | None | HT, HL, migraine | Left body | 72 | Normal | 50 |
| 36 | 62 | F | 13 | Days | During worship | None | HT, HL | Right body | 11 | Right frontotemporoparietal hypoperfusion | 33 |
| 37 | 59 | F | n/a | n/a | n/a | n/a | n/a | Right body | 26 | Normal | 168 |

Abbreviations: M = male; F = female; HT = hypertension; HL = hyperlipidemia; DM = diabetes mellitus; n/a = not available.

^a^When patients were able to explain the reasons for their hospitalizations and to form new memories, we considered their episode of TGA to be over.
