# Supplementary material for: Reflections of Two Parallel Pathways between the Hippocampus and Neocortex in Transient Global Amnesia: A Cross-Sectional Study Using DWI and SPECT
Source: PLoS One. 2013 Jul 5;8(7):e67447. doi: 10.1371/journal.pone.0067447 (PMC3702497; doi:10.1371/journal.pone.0067447)
Supplement: Table S2 — Comparison of baseline characteristics between the included and excluded patients. (DOC) [file pone.0067447.s003.doc]

**Table S2. Comparison of baseline characteristics between the included and excluded patients.**

|  | **Included patients^a^**  **(n = 37)** | **Excluded patients**  **(n = 51)** | **P value^b^** |
| --- | --- | --- | --- |
| **Age in years, mean (SD)** | 59.35 (7.30) | 62.04 (11.51) | 0.215 |
| **Men** | 11 (29.7%) | 23 (45.1%) | 0.144 |
| **Precipitating factor** |  |  |  |
| **Physical stress** | 17 (47.2%) | 15 (29.4%) | 0.090 |
| **Emotional stress** | 17 (47.2%) | 19 (37.3%) | 0.353 |
| **Vomiting** | 5 (13.9%) | 2 (3.9%) | 0.121 |
| **Duration of TGA in hours, mean (SD)** | 7.50 (4.47) | 5.33 (3.73) | 0.016 |
| **Associated symptoms** |  |  |  |
| **Headache** | 12 (33.3%) | 10 (19.6%) | 0.147 |
| **Dizziness** | 4 (11.1%) | 0 | 0.026 |
| **Nausea** | 5 (13.9%) | 1 (2.0%) | 0.078 |
| **None** | 20 (55.6%) | 38 (74.5%) | 0.065 |
| **Hypertension** | 11 (30.6%) | 20 (39.2%) | 0.406 |
| **Diabetes** | 3 (8.3%) | 3 (5.9%) | 0.657 |
| **Hyperlipidemia** | 13 (36.1%) | 17 (33.3%) | 0.788 |
| **Migraine** | 3 (8.3%) | 4 (7.8%) | >0.999 |

Values are number (%) unless indicated.

Abbreviations: TGA = transient global amnesia.

^a^For one patient from the included patients, only data concerning age and gender were available

^b^P values were obtained using Student’s *t*-test, Pearson’s chi-square test or Fisher’s exact test as appropriate.
