# Supplementary material for: Reflections of Two Parallel Pathways between the Hippocampus and Neocortex in Transient Global Amnesia: A Cross-Sectional Study Using DWI and SPECT
Source: PLoS One. 2013 Jul 5;8(7):e67447. doi: 10.1371/journal.pone.0067447 (PMC3702497; doi:10.1371/journal.pone.0067447)
Supplement: Table S3 — Coefficient loadings of variables for each dimension in MFA (multiple factorial analysis). (DOC) [file pone.0067447.s004.doc]

**Table S3. Coefficient loadings of** variables for each dimension in MFA.

|  | Dimension 1 | Dimension 2 |
| --- | --- | --- |
| Hippocampal head lesion | 0.511 | 0.003 |
| Hippocampal body lesion | 0.458 | 0.003 |
| Hippocampal tail lesion | 0.003 | 0.017 |
| Men | 0.208 | 0.063 |
| Women | 0.208 | 0.063 |
| Age <60 years | 0.094 | 0.609 |
| Age ≥60 years | 0.094 | 0.609 |
| Precipitation by physical factor | 0.079 | 0.004 |
| Precipitation by emotional factor | 0.176 | 0.007 |
| Precipitation by vomiting | 0.288 | 0.339 |
| Headache | 0.039 | 0.007 |
| Dizziness | 0.304 | 0.064 |
| Nausea | 0.301 | 0.353 |
| No associated symptoms | 0.233 | 0.034 |
| Hypertension | 0.087 | 0.191 |
| Diabetes | 0.144 | 0.011 |
| Hyperlipidemia | 0.127 | 0.194 |
| Vascular risk factors | 0.294 | 0.156 |
| Migraine | 0.090 | 0.003 |
| Percent of total variance | 19.677% | 14.323% |

Abbreviations: MFA = multiple factorial analysis.
